# Association of clinical laboratory parameters with latent tuberculosis infection among healthcare workers of primary health centers―A cross-sectional observational study

**DOI:** 10.1371/journal.pgph.0004873

**Published:** 2025-06-27

**Authors:** Sivaprakasam T. Selvavinayagam, Ganga Sankar, Yean K. Yong, Sathish Sankar, Ying Zhang, Hong Y. Tan, Pachamuthu Balakrishnan, Amudhan Murugesan, Manivannan Rajeshkumar, Asha Frederick, Masilamani Senthil Kumar, Paulkanraj PriyaRaj, Jesudoss Prabhakaran, Pattusamy Sangeetha, Pasupathy Arunpathy, Rajamani Charu, Nagarajan Muruganandam, Deepak M. Sakate, Deepak Jayakumar, Prabu Dhandapani, Parthiban Rudrapathy, Vijayakumar Velu, Marc Emmenegger, Marie Larsson, Esaki M. Shankar, Sivadoss Raju

**Affiliations:** 1 Directorate of Public Health and Preventive Medicine, DMS Campus, Chennai, Tamil Nadu, India; 2 State Public Health Laboratory, Directorate of Public Health and Preventive Medicine, DMS Campus, Chennai, Tamil Nadu, India; 3 Laboratory Center, Xiamen University Malaysia, Sepang, Selangor, Malaysia; 4 Kelip‐kelip! Center of Excellence for Light Enabling Technologies, Xiamen University Malaysia, Sepang, Selangor, Malaysia; 5 Department of Microbiology, Center for Infectious Diseases, Saveetha Dental College and Hospitals, Saveetha Institute of Medical and Technical Sciences, Saveetha University, Chennai, Tamil Nadu, India; 6 Chemical Engineering, Xiamen University Malaysia, Sepang, Malaysia; 7 School of Traditional Chinese Medicine, Xiamen University Malaysia, Sepang, Malaysia; 8 Department of Research, Meenakshi Academy of Higher Education and Research (MAHER) (Deemed to be University), Chennai, Tamil Nadu, India; 9 Department of Microbiology, Government Theni Medical College and Hospital, Theni, Tamil Nadu, India; 10 Indian Council Medical Research - Regional Medical Research Center, Sri Vijaya Puram (Port Blair), Andaman and Nicobar Islands, India; 11 Department of Statistics and Applied Mathematics, Central University of Tamil Nadu, Thiruvarur, Tamil Nadu, India; 12 Department of Microbiology, Dr. ALM Post Graduate Institute of Basic Medical Sciences, University of Madras, Chennai, Tamil Nadu, India; 13 Microbiology Division, Department of Clinical Laboratory Services and Translational Research, Malabar Cancer Center (Post Graduate Institute of Oncology Sciences and Research), Thalassery, Kerala, India; 14 Department of Pathology and Laboratory Medicine, Emory University School of Medicine, Division of Microbiology and Immunology, Emory National Primate Research Center, Emory Vaccine Center, Atlanta, Georgia, United States of America; 15 Institute of Neuropathology, University Hospital Zurich, Zürich, Switzerland; 16 Division of Medical Immunology, Department of Laboratory Medicine, University Hospital Basel, Basel, Switzerland; 17 Division of Molecular Medicine and Virology, Department of Biomedical and Clinical Sciences, Linköping University, Linköping, Sweden; 18 Infection and Inflammation, Department of Biotechnology, Central University of Tamil Nadu, Thiruvarur, Tamil Nadu, India; University of California Irvine, UNITED STATES OF AMERICA

## Abstract

Healthcare workers (HCWs) are at high risk of tuberculosis (TB) infection due to their continued occupational exposure to patients with active TB disease. The prevalence of latent TB infections (LTBI) among the HCWs of primary healthcare centers (PHCs) has seldom been investigated. PHCs provide effective and preventive medical care largely for the rural population. Comparatively, the HCWs of PHCs are likely to have an increased risk of occupational exposure and reactivation of LTBI. A cross-sectional study (March–April 2024) was carried out to assess the prevalence of LTBI among the HCWs of 64 PHCs across Thiruvallur district, India. Blood samples (n = 392) were analyzed using a commercial QuantiFERON-TB Gold Plus assay. A comprehensive hematological, biochemical, and immunological workup was performed, including cell count, blood glucose determination, liver/renal function tests, and serum ferritin concentration estimation, which were subsequently correlated with LTBI status using multivariate logistic regression analysis. The study revealed an LTBI prevalence of 25.3% (n = 99) among HCWs of PHCs. The red cell distribution width (RDW) was significantly associated (p = 0.002) with LTBI positivity among the different parameters analyzed. Factors such as individuals’ age (p = 0.029), underlying comorbid conditions (30.3%; p = 0.035), and longer employment duration (28%; p = 0.034) were significantly associated with IGRA positivity. Further, IGRA positivity was significantly associated with decreased RDW standard deviation (RDW-SD). This phenomenon was observed especially among females, the obese, and participants with the ‘O’ blood group. Although the exact prevalence of LTBI in the general population is not known, it is estimated to range from 20-48%. The study reported the prevalence of LTBI among HCWs of PHCs (25.3%) and factors associated with IGRA positivity including age, underlying comorbid conditions, and years of employment. Our findings will aid in developing and establishing an appropriate framework for TB screening and clinical testing guidance for HCWs.

## Introduction

Pulmonary tuberculosis (TB) predominates among the infectious causes of death especially in low- and middle-income countries despite the availability of effective diagnostic tools and anti–tubercular therapeutic (ATT) drugs. The WHO Global Tuberculosis Report 2024 estimated 10.8 million TB cases in 2023, which is tantamount to 134 incident cases per 100 000 population, with most cases (45%) documented from across South-East Asia. The WHO framework with the *End-TB Flagship Strategy* was initiated to reduce TB incidence and deaths [1]. However, the COVID-19 pandemic appears to have disrupted the critical components of the End–TB strategy – *Find and Treat All*. A significant reduction (~18%) of newly diagnosed TB was reported in 2020, while a surge in cases and deaths was noticed during 2021–22 [2]. Several respiratory infections caused by different viral and bacterial pathogens over an individual’s lifetime compromise lung function and predispose to the reactivation of other latent infections. For example, the respiratory system shares the battlefield for both SARS-CoV-2 and *M. tuberculosis* (MTB), which can trigger lung damage [3]. The increase in TB incidence and deaths post-COVID-19 could be attributed to a perturbed immune system and subsequent reactivation of latent TB infection (LTBI) [4–8]. It has been estimated that one–third of the global population is latently infected by TB, and about 5–10% of the latently infected, reactivate within two years of primary infection [9]. The updated “WHO Consolidated Guidelines on Tuberculosis Management” is focused on global populations at risk of developing TB to curtail the transmission chain of TB. Reactivation of LTBI adds to ongoing active transmission for new active TB cases, and HCWs have a high risk of infection compared to the general population. Reactivation among HCWs and subsequent transmission in both healthcare settings, as well as community settings, poses a formidable challenge to TB prevention and treatment. This underscores the importance of screening for LTBI among HCWs to render effective TB elimination [10].

The pooled prevalence of LTBI among HCWs globally ranges from 1–85% based on several systematic reviews and meta-analysis [11–15]. Nonetheless, in some countries, the prevalence among HCWs of PHCs who own the highest risk of progression to active TB disease and transmission largely remains unknown. Certain underlying comorbidities including renal and liver diseases, malnutrition, and prolonged exposure to TB-infected individuals are likely to increase the risk of transition of LTBI into clinical TB disease [16–18].

LTBI detection by tuberculin skin test (TST) suffers several pitfalls including higher turn-around-time, false-positivity due to the cross-reaction with the vaccine, and other bacterial antigens. It also suffers from poor sensitivity in immunocompromised subjects and in differentiating active disease from latent infections [19–21]. Implementation of guidelines for TB prevention among HCWs has been one of the top priorities of the National TB Elimination Program (NTEP), a flagship program of the Government of India. Therefore, it is imperative to identify and treat appropriately to achieve the goal of ending TB by 2030. Here, a cross-sectional study was carried out on the prevalence of LTBI among primary HCWs in the Thiruvallur district, India. To investigate factors associated with LTBI, a comprehensive analysis of socio-demographic details, associated risk factors, and clinical and laboratory investigations was undertaken.

## Materials and methods

### Study design

A cross-sectional observational study was carried out on the HCWs including physicians, nurses, pharmacists, laboratory technicians, different categories of nurses that include staff nurses who primarily work at the hospitals and village health nurses who primarily work in the community, and other hospital workers (health assistants and housekeepers) from all the 64 primary health centers (PHCs) of Thiruvallur district, who maintained close contact with patients with confirmed or unknown TB disease status. The start date of the project was 08^th^ May 2024 and the end date was 7^th^ June 2024. Individuals aged ≥18 years, who consented to participate with no clinical evidence of TB were included, while individuals who were <18 years of age, pregnant, HIV infected, and/or with a history of active or latent TB infection were excluded.

**Fig 1:**
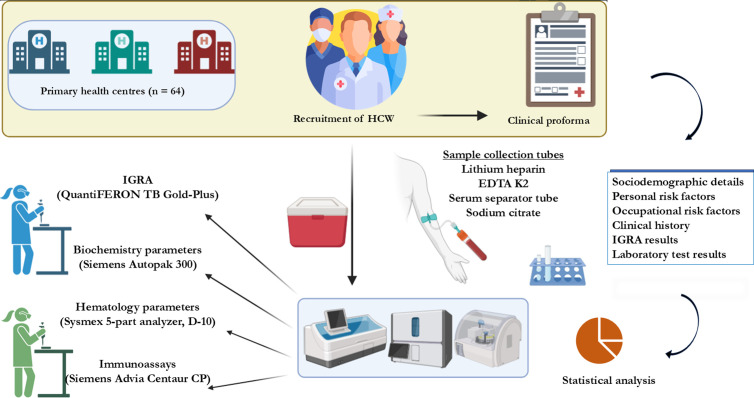
Schematic flowchart of the study.

A total of 489 individuals were approched, of which 392 (80.2%) individuals meeting the inclusion criteria and who consented for the study were recruited. A detailed clinical proforma on clinical history, comorbidities, and risk factors together with sociodemographic details were collected from each participant (Fig 1).

### Ethical approval

This study was performed within the ethical standards of the Declaration of Helsinki. The study was approved by the Institutional Ethical Committee (Approval Reference number: DPHPM/IEC/010/V2 Dated 20/03/2024) of the Directorate of Public Health and Preventive Medicine, Government of India. Participants provided written informed consent for the study.

### Inclusivity in global research

Additional information regarding the ethical, cultural, and scientific considerations specific to inclusivity in global research is included in the Supporting Information (S1 Checklist).

### Clinical specimens

Blood samples in lithium heparin tubes (5 mL), gel tubes (3 mL), EDTA tubes (2 mL), and citrate tubes (1.6 mL) were collected by venipuncture. The block PHC located in every administrative block (administrative sub-divisions of the local regional government in India) of the district was selected as a collection hub from where the samples were transported in the cold chain to the State Public Health Laboratory (SPHL), Directorate of Public Health and Preventive Medicine within two hours of collection. All the samples were promptly processed for hematological, biochemical, and immunological analyses. Appropriate quality controls were included for all the diagnostic assays.

### Hematological and biochemical parameters

The erythrocyte sedimentation rate (ESR) by Westergren method and complete blood count (CBC) were analyzed using a 5-part hematology analyzer (XN-550, Sysmex). The test parameters included total WBC count, RBC count, hemoglobin, hematocrit (HCT), mean corpuscular volume (MCV), mean corpuscular hemoglobin (MCH) and its concentration (MCHC), platelet count (PLT), RDW (RDW-SD and RDW-CV), platelet distribution width (PDW), mean platelet volume (MPV), platelet large cell ratio (P-LCR), and plateletcrit (PCT). In addition, differential counts and percentages of neutrophils, lymphocytes, monocytes, eosinophils, and basophils were also assessed. The mean cell size and the breadth of the distribution curve were used to calculate the RDW-CV. The standard deviation (SD) of the mean cell size was divided by MCV to derive the percentage values. The RDW-CV typically ranges between 11 and 15%. Biochemical tests including liver function test, renal function test, C-reactive protein (CRP) and glycosylated hemoglobin (HbA1C) as a measure of glucose levels, were carried out using an automated Autopak 300 clinical chemistry analyzer (Siemens) and a D-10 hemoglobin testing system (Bio-Rad).

### Interferon-gamma release assay (IGRA)

The blood samples were processed for IGRA testing using a commercial QuantiFERON TB-Gold plus assay (Qiagen) as per the manufacturer’s instructions. The test employed a peptide cocktail simulating mycobacterial proteins to stimulate lymphocytes in the heparinized whole blood. The values are expressed in IU/mL. A TB1/TB2 value (minus Nil) of ≥0.35 and ≥25% of Nil was considered positive and reported as a potential LTBI positive status.

### Ferritin assay

Serum ferritin concentration was determined, and was expressed as ng/µL. The kit utilized a two-site sandwich chemiluminescence method and had an assay range of 0.5–1650 ng/µL. The normal reference range for males was 22–332 ng/µL and for females was 10–291 ng/µL as recommended by the assay kit.

### Statistical analysis

The primary aim was to report the prevalence of LTBI among HCWs of PHCs. The association between the LTBI positivity and the sociodemographical and clinical characteristics of the participants (listed in Table 1) was analyzed using the Chi-square test; while the association between the LTBI positivity and clinical laboratory parameters (e.g., hematological, renal, liver, glucose parameters) were analyzed using the Mann-Whitney U test. To improve comparability between biomarkers with different scales, the level of each biomarker was transformed into a uniform scale, the Z-score. Biomarkers were analyzed together using a standard Z-score. Each biomarker was first transformed to Z-score using the following formula, z=(x–μ)/σ, where (x) = subject’s biomarker levels, (μ) = mean of the biomarker levels, and (σ) = standard deviation biomarker levels. The predictive power of biomarkers in aiding the prediction of LTBI-positivity was examined using receiver operating characteristic (ROC) analysis. ROC analysis was performed between IGRA-positive and -negative participants to assess the suitability of RDW-SD, RDW-CV%, and eosinophil percentage as surrogate markers of LTBI. Fold change was done by normalizing the levels of a biomarker in the IGRA-positive group against the median level of the same biomarker in the LTBI-negative group, and the biomarker with significant fold changes was plotted in a heatmap according to the colour scheme indicated. Logistic regression analysis was performed univariately to identify biomarkers that were significantly associated with the LTBI-positivity and plotted as volcanic plots. The biomarkers that showed significant association in the univariate were regarded as candidate predictors and were included in multivariate regression analysis. The odds ratio (OR) and 95% confidence interval (CI) were estimated. Statistical analyses were performed using PRISM software, ver.5.02 (GraphPad Software, San Diego, CA). Logistic regression was performed using SPSS software, ver.20 (IBM, USA). A p-value of ≤0.05 was considered to be significant. We did not adjust the resulting p values for multiple comparisons.

**Table 1:** Socio-demographic and clinical characteristics of IGRA positive individuals.

Socio-demographic and clinical characteristics	Total sample = 392 N (%)	IGRA, N (%)		P-value
		Positive	Negative	
**Gender**				
Male	94 (24)	27 (27.3)	67 (22.9)	0.375
Female	298 (76)	72 (72.7)	226 (77.1)	–
**Co-morbidities**				
Asthma	13 (3.3)	3 (3)	10 (3.4)	0.0349
Thyroid disorders	10 (2.5)	5 (5)	5 (5)	–
Diabetes	48 (12.2)	15 (15.2)	33 (11.3)	–
Hypertension	27 (6.9)	8 (8.1)	19 (6.5)	–
Autoimmune disease	3 (0.8)	1 (1)	2 (0.7)	–
None	289 (73.7)	65 (65.7)	224 (76.5)	–
**Domiciliary status**				
Rural	241 (61.5)	67 (67.7)	174 (59.4)	0.143
Urban	151 (38.5)	32 (32.3)	119 (40.6)	–
**BMI**				
Underweight	14 (3.6)	2 (2)	12 (4.1)	0.248
Normal	125 (31.9)	32 (32.3)	93 (31.7)	–
Obese class 3	101 (25.8)	32 (32.3)	69 (23.5)	–
Overweight	152 (38.8)	33 (33.3)	119 (40.6)	–
**BCG**				
Vaccinated	357 (91.1)	89 (89.9)	268 (91.5)	0.557
Unknown	28 (7.1)	9 (9.1)	19 (6.5)	–
Not vaccinated	7 (1.8)	1 (1)	6 (2)	–
**Education**				
Primary	40 (10.2)	13 (13.1)	27 (9.2)	0.415
Secondary	87 (22.2)	24 (24.2)	63 (21.5)	–
Graduate	236 (60.2)	53 (53.5)	183 (62.4)	–
Postgraduate	29 (7.4)	9 (9.1)	20 (6.8)	–
**Work experience**				
Less than a year	39 (9.9)	6 (6.1)	33 (11.3)	0.047
2–5	128 (32.7)	25 (25.3)	103 (35.1)	–
6–10	103 (26.3)	34 (34.3)	69 (23.5)	–
More than 10 years	122 (31.1)	34 (34.3)	88 (30)	–
**TST**				
Done	20 (5.1)	6 (6.1)	14 (4.8)	0.616
Not done	372 (94.9)	93 (93.9)	279 (95.2)	–
**Treated TB case**				
1 to 5 cases	54 (13.8)	9 (9.1)	45 (15.3)	0.482
6 to 10 cases	27 (6.9)	7 (7.1)	20 (6.8)	–
More than 10 cases	100 (25.5)	27 (27.2)	73 (24.9)	–
No	211 (53.8)	56 (56.5)	155 (52.9)	–
**HbA1C**				
Fair control	21 (5.4)	9 (9.1)	12 (4.1)	0.136
Good control	82 (20.9)	19 (19.2)	63 (21.5)	–
Normal	250 (63.8)	58 (58.6)	192 (65.5)	–
Poor control	39 (9.9)	13 (13.1)	26 (8.9)	–
**LFT**				
Abnormal	206 (52.6)	51 (51.5)	155 (52.9)	0.811
Normal	186 (47.4)	48 (48.5)	138 (47.1)	–
**RFT**				
Abnormal	99 (25.3)	19 (19.2)	80 (27.3)	0.108
Normal	293 (74.7)	80 (80.8)	213 (72.7)	–
**CRP**				
Abnormal	114 (29.1)	35 (35.4)	79 (26.9)	0.112
Normal	278 (70.9)	64 (64.6)	214 (73)	–
**Smoking**				
Yes	4 (1)	2 (2)	2 (0.7)	0.252
No	388 (99)	97 (97.9)	291 (99.3)	–
**Alcohol use**				
Yes	14 (3.6)	4 (4)	10 (3.4)	0.771
No	378 (96.4)	95 (95.9)	283 (96.6)	–
**Anti-SARS-CoV-2 IgG**				
Reactive	390 (99.5)	98 (98.9)	292 (99.6)	0.419
Non–reactive	2 (0.5)	1 (1)	1 (0.3)	–
**Ferritin**				
Abnormal	57 (14.5)	13 (13.1)	44 (15)	0.645
Normal	335 (85.5)	86 (86.9)	249 (84.9)	–

## Results

### Sociodemographic and clinical characteristics of study participants

**Fig 2:**
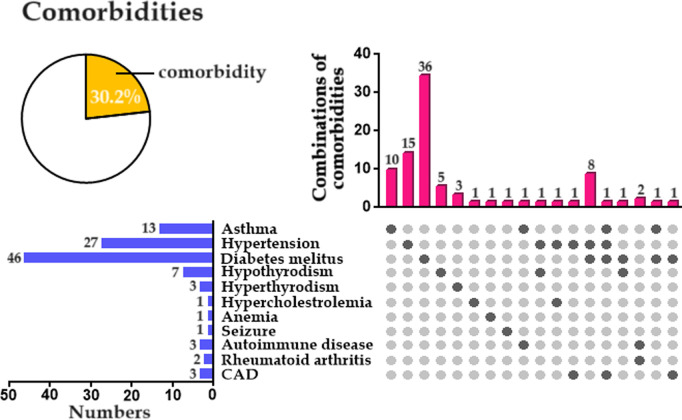
Comorbidities among LTBI-positive individuals. CAD: Coronary Artery Disease.

A total of 392 individuals between between 08 May 2024 and 07 June 2024 were included. A detailed clinical proforma was collected from each participant to assess the associated risk factors, and comprehensive laboratory investigations were carried out from the blood samples. The participants from each PHC included different categories of HCWs. Among these, other hospital workers (health assistants and housekeepers) were higher in number (n = 174; 44.4%) followed by nurses (n = 149; 38.0%), and physicians (n = 69; 17.6%). The age of the participants ranged from 23 to 60 years with a median of 38 years; The number of female participants was 298 (76%) and male participants was 94 (24.0%). Among nurses, 109 were female (93.0%), among physicians 40 were female (58.0%), among pharmacists, 16 were female (53.0%), and among laboratory technicians, 52 were female (89.6%) (Fig 2).

### Prevalence of LTBI among healthcare workers

Although the exact prevalence of LTBI in the general population is not known, it is estimated to range from 20 to 48%. In our study, among the 392 samples tested, 99 (25.3%) revealed a positive IGRA test. Among the different categories of HCWs tested, village health nurses showed the highest positive rate (38.0%) followed by pharmacists (33.0%), other hospital workers (32.0%), and staff nurses (25.0%). The percentage of IGRA positives among males (28.7%) did not differ significantly from females (24.2%, p = 0.414). The domiciliary status of the study participants among the IGRA-positives was also analyzed. Although participants from rural were higher in numbers (61.5%) compared to urban areas, the percentage of IGRA-positive cases in rural and urban populations were 27.8% and 21.2%, respectively, and the association between the domiciliary status and IGRA-positive rate was not statistically significant (p = 0.153).

Measurement of body mass index (BMI) among the 392 participants indicated that a larger proportion were overweight (38.8%), 3.6% were underweight, and 31.9% showed normal BMI. Intriguingly, 25.8% of individuals were identified with class III obesity. The number of HCWs with positive IGRA results was highest among class III obese participants (31.0%) followed by participants with normal BMI (25%). While a majority of the participants were vaccinated for Bacillus Calmette-Guérin (BCG) and were aware of their vaccination status (91.1%), 7.1% were unfamiliar with their vaccination history, and 1.8% had not been vaccinated. The number of IGRA positives was higher in the population with unknown vaccination status (32.1%) followed by the vaccinated population (24.9%). Among the HCWs, 60.2% were graduates and 22.2% had completed secondary education. However, educational categories did not show any difference between IGRA positives and negatives. Of the 392 participants, n = 21 (5.4%) disclosed a fair control of blood glucose with a mean HbA1c value of 7.4 and a mean blood glucose level of 166 mg/dL. Among the 392 participants, 52.6% (n = 206) had abnormal LFT and 25.3% (n = 99) had abnormal RFT (Table 1). No statistically significant difference was observed between the two groups. SARS-CoV-2 IgG was detected among all participants except two, of which one was positive for IGRA and the other was negative.

Participants with a positive LTBI status were offered prophylactic treatment through the authorized TB elimination program, and the results were updated in the NIKSHAY portal (a patient management system dedicated for TB under the National Tuberculosis Elimination Program of the Ministry of Health and Family Welfare, Government of India).

### Factors associated with IGRA positivity

Among different demographic markers analyzed, the age of the participants and duration of employment were significantly associated with IGRA positivity (p = 0.029 and p = 0.034, respectively); however, gender and BMI were not significantly associated. Interestingly, neither TB exposure history nor the number of TB cases treated by the HCW had a significant association with LTBI positivity indicating a possible acquisition of infection from the hospital but may be indirectly from treated patients.

**Fig 3:**
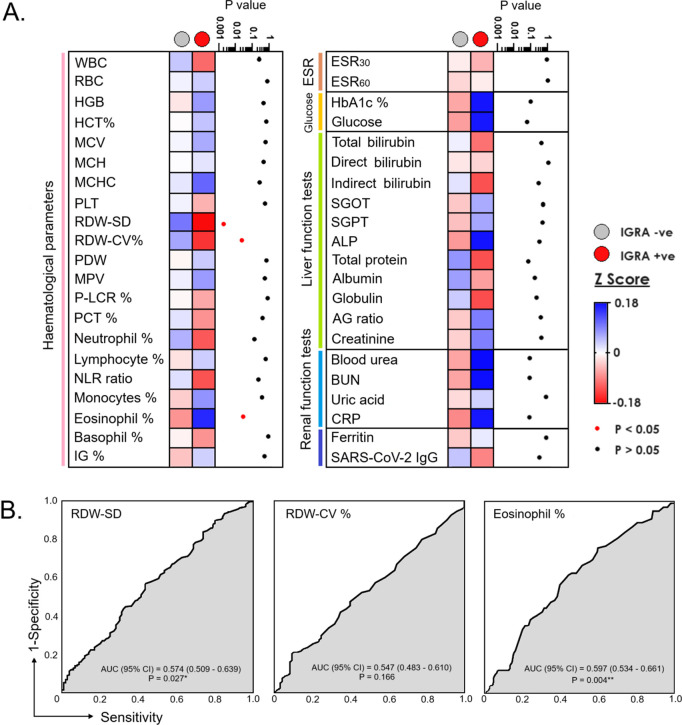
(A) Comparison of the standardized levels (Z-score) of parameters among HCWs with IGRA+ve and IGRA-ve (B) Receiver operating characteristic (ROC) curves for prediction of latent tuberculosis infection (LTBI) using RDW-SD, RDW-CV%, and eosinophils %; AUC = Area under curve. * and ** represent *p < *0.05, and *<*0.01, respectively.

When compared between IGRA-positives and -negatives, the positives showed a statistically significant lower Z-score in RDW-SD, RDW-CV% (p = 0.002 and p = 0.026, respectively), and had a higher Z-score in eosinophil percentage (p = 0.031) (Fig 3A). The diagnostic efficacy of RDW-SD and eosinophil percentage for diagnosis of LTBI was only marginal, where the AUC of 0.574; p = 0.002 and AUC = 0.597; p = 0.004, respectively. The AUC for RDW-CV% was not significant (Fig 3B).

**Fig 4:**
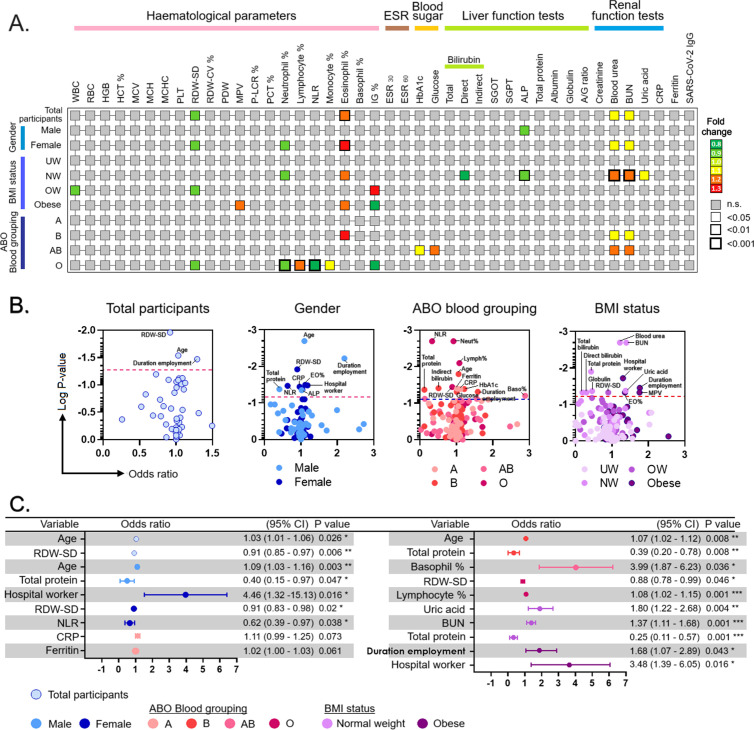
Parameters associated with LTBI among HCWs stratified by gender, BMI status, and ABO blood group. (A) Comparison of fold-change of parameters and their association with LTBI, (B) Univariate logistic regression analysis of parameters associated with LTBI, (C) Multivariate logistic regression analysis of parameters, associated with LTBI (The Hosmer–Lemeshow value for this model was P = 0.162). UW: underweight; NW: normal weight; OW: overweight; NLR: neutrophil-to-lymphocyte ratio; EO%: Eosinophil count; Baso%: Basophil count.

Consummated from our previous studies on biomarkers such as plasma CXCL8 and MCP-1 that aid in diagnosis and as surrogate predictors of disease progression [22] and platelet-large cell ratio and ESR [23] that were probably affected by demographics, we here measured the fold change of each biomarker (normalized against IGRA-negative) and stratified by gender, ABO blood grouping, and BMI status. Biomarkers that showed significant alteration in fold change were indicated according to the colour scheme (Fig 4A). The results showed that the levels of biomarkers were differentially altered in gender, BMI status, and ABO blood grouping. The results showed that IGRA positivity was significantly associated with decreased RDW-SD in females, overweight, and participants with the ‘O’ blood group. Eosinophil percentage was significantly associated with IGRA positivity in females, participants with normal weight and obesity as well as participants with blood group type ‘B’. Participants with blood group ‘O’ showed the most profound alteration in hematological markers, where IGRA positivity was associated with decreased percentages of neutrophils and immunoglobulin, as well as increased percentages of lymphocytes and monocytes. Besides, participants with IGRA positivity also showed elevation of blood urea and BUN in total participants, females, participants with normal weight as well as participants with blood groups B and AB.

### Multivariate analysis of factors associated with IGRA positivity

To identify the biomarker that is independently associated with IGRA-positivity, we performed logistic regression analysis whereby each biomarker was tested univariately against IGRA-positive, and biomarkers with p < 0.05 were regarded as candidates (Fig 4B). The candidates were then subjected to a multivariate logistic regression analysis and biomarkers with p* < *0.05 were considered as independent predictors in their respective models (Fig 4C). We found that age was significantly associated with increased odds of IGRA-positivity, whereby every increase of age by one year was associated with increased odds of having IGRA-positive by 3.0%, (95% CI = 1–6%); p = 0.026. Whilst every decrease of RDW-SD by one unit was significantly associated with decreased odds of IGRA-positive status by 9%, (95% CI = 3–15%); p = 0.006.

Considering age, BMI, and ferritin as the confounding factors for CRP, logistic regression analysis indicated a significant association of age-adjusted CRP (p = 0.026) and age-adjusted ferritin (p = 0.032) with LTBI-positive individuals. The univariate and multivariate logistic regression analyses on each parameter were carried out (Fig 4C). Spearman’s correlation (rho) was used to evaluate the relationship between the biomarkers and determine whether the biomarkers are correlated. The correlations were considered significantly strong if rho was ≥ 0.60 and p was < 0.05. The study did not show any correlation between the biomarkers in the logistic regression model (see S1 Fig).

## Discussion

We carried out a cross-sectional, observational study to estimate the prevalence of LTBI among HCWs of primary health centers across the Thiruvallur district, India. The present study identified a prevalence of 25.3% among the HCWs of PHCs. Among the different categories of HCWs, the highest positivity rate was observed among village health nurses followed by other workers including staff nurses and physicians.

For effective control of TB, appropriate identification and treatment of latently infected individuals are vital to reduce their risk of progression to active disease, especially among the high and moderate risk groups. PHCs in India act as the frontline in preventive medical care for the rural population and provide affordable primary healthcare services including laboratory diagnosis and treatment towards universal health coverage [24]. HCWs are considered to be the moderate-risk group for TB reactivation. Other factors such as malnutrition, immune suppression or weakened immune system, smoking, and comorbidities such as diabetes, thyroid dysfunction, renal dysfunction, and liver dysfunction may increase the risk substantially [25–30]. In addition, increased tuberculosis is contributed by social or environmental determinants and personal health determinants which are often ignored while assessing the prevalence of LTBI in the community. Overall, this puts the determination of universal health coverage in jeopardy when the fundamental healthcare system at the primary level is affected. In our study, we determined to study the prevalence of LTBI among HCWs of PHCs and assess their LTBI-associated laboratory parameters using multiple logistic regression analysis.

Our previous investigation on household contacts of active TB patients indicated a 43% positive rate with a female predominance [22]. It is therefore quintessential to identify LTBI test-positive individuals who act as reservoirs and seedbeds for transmission of reactive TB disease in the community. Likewise, HCWs have a very high occupational risk of reactivation of latent infection [31]. However, there are very limited reports available on the HCWs especially from PHCs. Hence, it is of paramount importance for frontline healthcare providers in primary healthcare settings to undertake periodical clinical and radiological self-screening and self-monitoring of fatal infections like tuberculosis. This is the first study to our knowledge that reports the prevalence of LTBI and associated risk factors among HCWs of primary health centers. The present study reported 25.3% positivity similar to trends seen among other risk groups such as household contacts of active TB patients [23]. Apriani *et. al.* reported a 16.2% prevalence of IGRA-positivity among medical and nursing students of Bandung, Indonesia [32], while Rafiza et. al. [33] reported a 10.6% prevalence of LTBI among HCWs in Malaysia. A systematic review of studies conducted in low- and middle-income countries (LMICs) indicated the association of prevalence and incidence of LTBI with years of work, work area and work category, and contact with active TB patients among HCWs. Overall, the prevalence of IGRA-positivity ranged from 9-86%, with a mean of 39% [15]. India being a low- and middle-income country, the prevalence of LTBI among HCWs in the Thiruvallur district reported herein was lower than average. A systematic review and meta-analysis on the prevalence of LTBI among HCWs of Latin American and Caribbean regions indicated a 35.3% prevalence of LTBI and was associated with long years of service, and exposure to TB patients and their family members. The study also indicated that professionals including physicians, nurses, and technicians had an increased risk of LTBI [34]. A meta-analysis conducted across Asian countries indicated a prevalence of 21% (by IGRA test), which substantiates a higher risk of developing LTBI, likely owing to extended occupational exposure [35]. However, the study did not include high TB burden countries including India, Pakistan, Nepal, and Bangladesh.

LTBI prevalence has been shown to increase with increasing duration of exposure [36]. In our study, we observed LTBI positivity to be associated with age and years of service (with both factors in collinearity) but not with TB exposure history or the number of TB cases treated by the HCW. This suggests the possible acquisition of infection from the hospital environment rather than directly from TB patients. A similar observation was also reported where medical school students in Indonesia who had direct contact with family or TB patients were negatively associated with IGRA positivity [32].

Despite the limitations, the IGRA test is widely used in diagnosis, to differentiate between active and latent infection, and to decide on initiating preventive therapy. IGRA test could remain positive long after treatment. Individuals who show a positive IGRA result may show a positive result throughout life despite the use of anti-tubercular drugs. Therefore, it is important to evaluate IGRA results carefully considering the patient’s past clinical history. Of our test population (n = 392), none had any history of TB disease and a total of 20 (5.1%) had a history of TST done previously. Based on their self-declaration, all were negative on testing although the exact timelines of their previous test are unknown. It is imperative to have a deeper understanding of the mode of pathogen transmission for better clinical management and to develop and institute appropriate public health policy.

Treating LTBI is of paramount importance to preventing progression to clinically active TB disease and could prevent drug resistance. However, before the decision whether to treat or not treat LTBI is made, it is important to assess the underlying conditions such as liver disease, alcohol use, pregnancy, and use of specific medications that could compromise liver function. Baseline laboratory liver function test and renal function test along with complete blood count were carried out to assess their health. Among 392 tested, 52% had abnormal liver function of which 25% of individuals were IGRA positive. Also, 25% had abnormal renal function, of which 19% were IGRA positive. Although the mean values of renal and liver biomarkers did not differ, the LTBI-positive individuals with abnormal LFT and/or RFT could have an increased risk of disease progression. Nevertheless, the baseline abnormal liver function tests are considered to be a major risk for drug-induced hepatotoxicity when treated for LTBI [37]. In our study group, 14 (3.6%) had a history of alcohol use, of these 11 continued to consume alcohol. Among the 14 alcohol users, four were positive for LTBI and all were current alcohol users. Alcohol use is a major risk factor for a weakened immune system and substantially increases the risk of reactivation [38,39].

Four individuals who were using immunosuppressive drugs, had no other risk factors and all of them were negative for LTBI. Various immunosuppressants including methotrexate, prednisolone, and phenytoin were being used by these individuals. The use of immunosuppressive drugs other than reactivation of latent TB has also been shown to impact the latent TB testing, however, IGRA tests are shown to be more sensitive than TST for immunosuppressed individuals. IGRA assay in contrast to skin testing, does not have a predetermined cut-off value and is specific to mycobacterial antigens. The proposed guideline indicates preventive treatment for LTBI without further testing for high-risk groups [40]. The prevalence of LTBI among pregnant women ranges from 14 to 48% in the USA and shows an increased risk of reactivation during the postpartum period [41]. There were no pregnant women in our study population; however, there were five lactating mothers and all five were negative for LTBI. IGRA assay is also reported to be more specific, sensitive, and unaltered by pregnancy [41].

Among the different hematological parameters tested including complete blood count, RDW-CD, RDW-SD, PDW, PCT, MPV, and platelet-large cell ratio, RDW-CV was found to be significant in LTBI-positive individuals. RDW, a measure of anisocytosis, is now considered a potential biomarker of chronic diseases. Factors such as persistent inflammation and inhibition of erythrocyte maturation induced by proinflammatory cytokines have been shown to cause increased RDW [42]. Although low RDW is considered clinically insignificant, the mechanism and role of the association of infections with altered RDW is still unclear [43]. The significance of hematological markers such as RDW-CV, RDW-SD, and inflammatory markers such as CRP have been shown to correlate with the severity and prognosis of different diseases including TB [44–47]. This is the first report on the use of RDW-CV and RDW-SD as significant markers indicative of probable association with LTBI status. Considering the cut-off limits of RDW-CV, among 99 LTBI positives, 15.0% had abnormal RDW and among 293 LTBI negatives, 24.0% had abnormal RDW. Its association and usefulness in differentiating LTBI should be further investigated with a large sample size and heterogenous test population. Likewise, ferritin level is an important factor when stratified with age, sex, CRP, and other inflammatory markers in determining the magnitude and distribution of iron deficiency and iron overload as a public health problem. Different studies indicated their role in predicting LTBI and prognosis [48,49]. Serum ferritin levels along with other inflammatory biomarkers such as CRP were found in two studies to indicate mycobacterial load and TB-associated inflammation during anti-tuberculous therapy [50,51]. Another study showed many markers including serum ferritin and certain risk factors not significantly associated with LTBI individuals [52]. Yet, iron metabolism indices could be further evaluated with different populations to evaluate its efficacy in differentiating LTBI from active disease and in prognosis.

Our study suffered from certain limitations, one of them is the study primarlly being focused on the HCWs of PHCs from one district in the state of Tamil Nadu, India, does not represent the true prevalence of LTBI or the burden of infection among HCWs. Nevertheless, this study is the first of its kind that highlights the prevalence of LTBI and associated risk factors among HCWs of PHCs in a district which could act as a model for future state/nationwide surveillance studies. Secondly, a comparison of the results of IGRA with TST or comparison with chest radiography among the study participants was not performed. However, the IGRA-positive study participants were followed up for further clinical evaluation and treatment.

## Conclusions

LTBI among HCWs, especially in resource-limited primary healthcare settings is of major global concern. The healthcare workforce striving towards ending TB is largely affected due to their occupational exposure risk. Hence, routine screening and appropriate medical interventions are crucial for the successful management of TB disease. There is a substantial lack of evidence on the prevalence of LTBI among HCWs in India, where the TB burden is high, especially in rural communities. The study showed an LTBI prevalence of 25% among HCWs of PHCs. The association of LTBI with individuals’ age, underlying comorbidities, and longer employment duration with LTBI positivity corroborates with previous reports and emphasizes the need for stronger health systems for effective TB disease prevention, treatment and rehabilitation. The study will help develop and implement an appropriate framework for tuberculosis screening and clinical testing guidance for HCWs. Coordinated scientific efforts and clinical strategies with appropriately aligned public health measures are urgently warranted for complete TB elimination.

## Supporting information

S1 Fig Spearman correlation analysis between the estimated biomarkers. (TIFF)

S1 Checklist S1_Checklist. (DOCX)
