# Supplementary figures and images for: Association of clinical laboratory parameters with latent tuberculosis infection among healthcare workers of primary health centers―A cross-sectional observational study
Source: PLOS Glob Public Health. 2025 Jun 27;5(6):e0004873. doi: 10.1371/journal.pgph.0004873 (PMC12204540; doi:10.1371/journal.pgph.0004873)

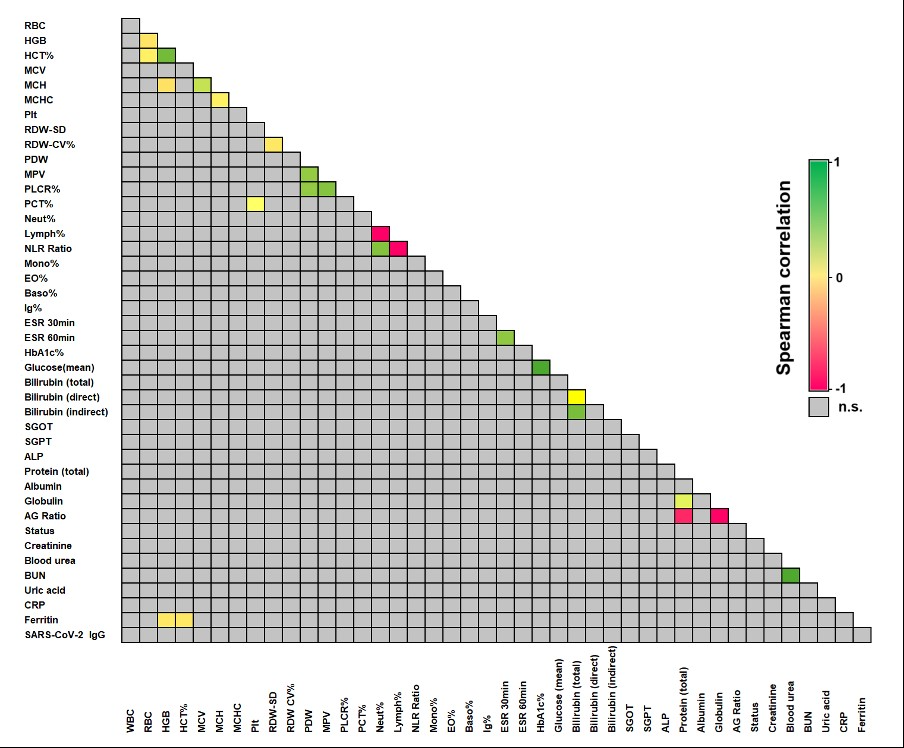

Supplement: S1 Fig — (TIFF) [file pgph.0004873.s001.tiff]
